# Miocene phytolith and diatom dataset from 10.3Myo diatomite formation, Fernley, Nevada, USA

**DOI:** 10.1016/j.dib.2023.109519

**Published:** 2023-08-23

**Authors:** Jacopo Niccolò Cerasoni, Megan C. O'Toole, Richa Patel, Yoel E. Stuart

**Affiliations:** a Department of Biology, Loyola University Chicago, 1050 W. Sheridan Rd., Chicago, IL 60626, USA; b Department of Chemistry, Loyola University Chicago, 1068 W. Sheridan Rd. Chicago, IL 60626, USA

**Keywords:** Palaeoecology, Botany, Phytoliths, Diatoms, Paleontology

## Abstract

Phytoliths are opal silica particles formed within plant tissues. Diatoms are aquatic, single-celled photosynthetic algae with silica skeletons. Phytolith and diatom morphotypes vary depending on local environmental and climatic conditions and because their silicate structures preserve well, the study of phytolith and diatom morphotypes can be used to better understand paleoclimatic and paleoenvironmental dynamics and changes. This article presents original data from an 820cm-deep stratigraphy excavated at the Hazen diatomite deposits, a high-elevation desert paleolake in the Fernley District, Northern Nevada, USA. The site has been studied for an assemblage of fossilized threespine stickleback, *Gasterosteus doryssus*, that reveal adaptive evolution. For this study, a total of 157 samples were extracted at 20 cm intervals covering approximately 24,500 years. After extraction, the samples were mounted on slides and viewed under 400-1000x light microscopy, enabling classification of 14 phytolith and 45 diatom morphotypes. Our data support paleoenvironmental reconstructions of the Hazen Miocene paleolake.

Specifications Table

**Table:** 

Subject	Ecology
Specific subject area	Palaeoecological reconstruction of Miocene paleolake local environment
Data format	Raw
Type of data	Table, Figure
Data collection	Sediment samples were extracted from Miocene paleolake diatomite deposits using metal chisels. The sediment was then ground with a needle tool in an 1.5ml eppendorf tube and mounted onto glass slides using a medium viscosity oil-based mounting agent and sealed with polymer nitrocellulose. Data was collected by brightfield optical microscopy using a Meiji MT4300L at 40x-100x magnification. Morphotypes were photographed using a Meiji Techno HD1500T camera. Morphotypes were identified via comparison to published literature.
Data source location	Institution: Loyola University Chicago • City/Town/Region: Chicago • Country: USA • Latitude and longitude for collected samples/data: -119.18379, 39.496 (WGS84)
Data accessibility	Repository name: Figshare Repository DOI: 10.6084/m9.figshare.22715866 Direct URL to data: https://figshare.com/articles/figure/Botanical_Microfossil_Morphotypes_-_Hazen_Diatomite_Formation/22715866

## 1 Value of the Data

• The fossil phytolith and diatom data can be used to reconstruct palaeoecological histories of local and regional vegetation, volcanic and fire activity, and other environmental variables.
• Explainable changes in abundance and composition of ancient microfossil communities may help predict how modern life might respond to similar environmental change.
• The paleoenvironment reconstruction may help explain observed adaptive evolution by the threespine stickleback fish (*Gasterosteus doryssus*) collected from the same stratigraphic sections.

## 2 Objective

**Fig. 1:**
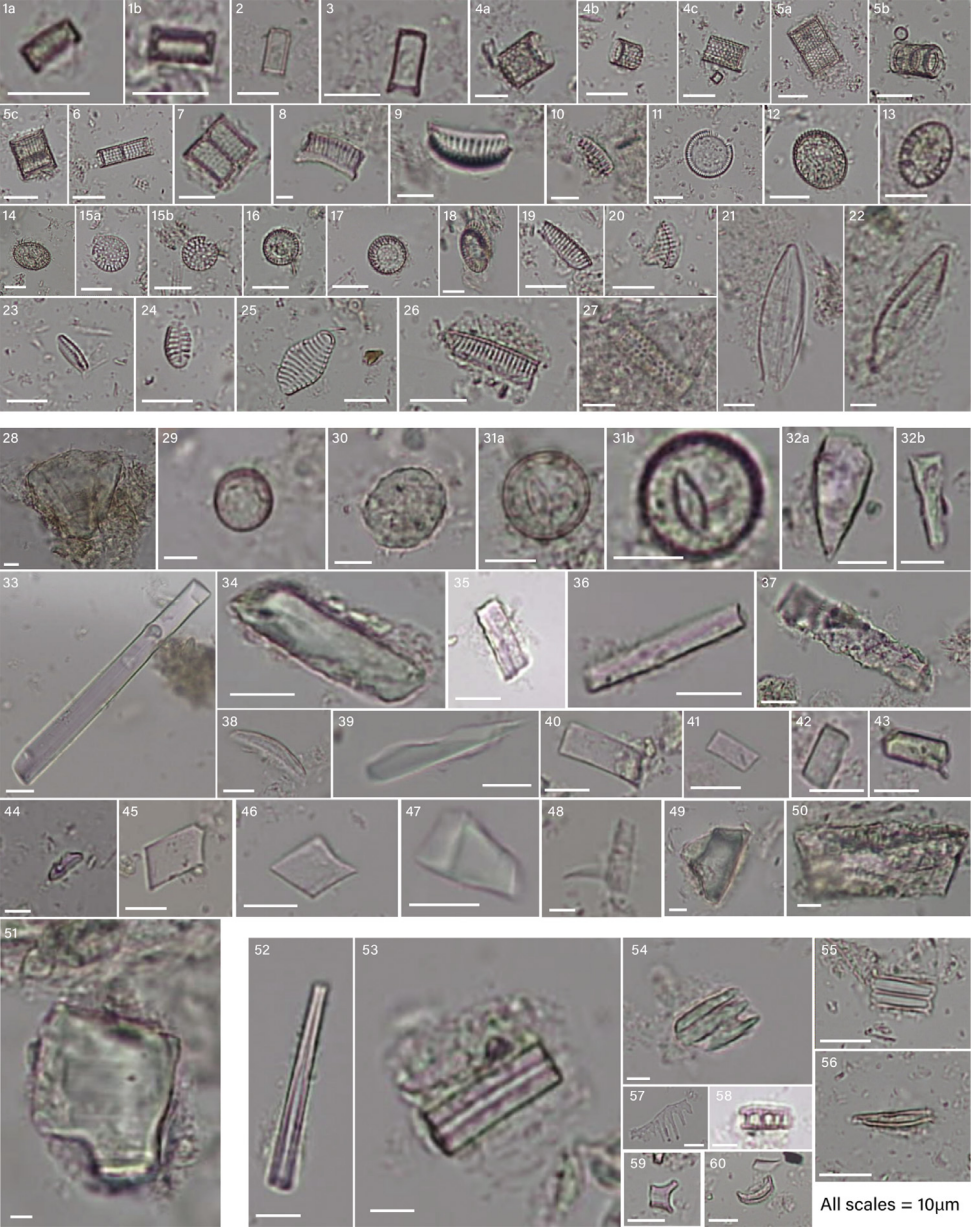
Paleontological microfossils, including diatoms, phytoliths, sponge spicules, and unidentified morphotypes. Diatoms: 1a-1b,2,3, unidentified centric; 4a-4c,6,7, Aulacoseira spp.; 5a-5c, Aulacoseira tenella; 8,10, Cymbella spp.; 9, Cymbella cymbiformis; 11,16, Lindavia rossii; 12,13,14,18, Pliocaenicus spp.; 15a-15b, Stephanodiscus spp.; 17, Cyclostephanos tholiformis; 19,20, Semiorbis spp.; 21,22, Craticula spp.; 23, Fragilariales spp.; 24, Geissleria spp.(?); 25, Punctastriata mimetica; 26, unidentified; 27, Aulacoseira subarctica. Phytoliths: 28, Poacae, Bulliform flabellate; 29,31a-31b, Broad-leaf/Conifer, Spheroid psilate; 30, Broad-leaf/Conifer, Spheroid ornate; 32a-32b, Panicoideae (?), Bilobate (fragmented); 33,34,35,36,39,43, unidentified, Elongate entire (Type 1); 37, unidentified, Elongate entire (Type 2); 38, unidentified, Elongate arcuate; 40,41,42,45,46, unidentified, Polygonal tabular; 44, Poacae, Rondel; 47,49, Broad-leaf/Conifer, Polyhedral (slereid); 48, Poacae, Acicular (hair-like); 49,50,51, Broad-leaf/Conifer, Blocky. Other: 52-53, sponge spicule fragments; 54-60, unidentified.

This article presents original phytolith and diatom data from a currently high-elevation desert paleolake in Northern Nevada (Fernley District, USA) comprised of Miocene diatomite [1,2]. The samples were originally collected to study the fossilized threespine stickleback, *Gasterosteus doryssus*. 157 samples spanning approximately 24,500 years of stratigraphical deposition were extracted following a published protocol [3] to identify diatom and phytolith morphotypes. The objective of this study was to offer a new dataset for future study of paleoenvironmental and paleoclimatic contexts of paleolakes from the Hazen Miocene. Micrographs and morphological and identification details of phytolith and diatom morphotypes can be found in Fig. 1 and Table 1.

**Table 1:** Phytolith, diatom and other microfossil morphotype descriptions and identifications.

Microfossil type	Morphology	Identification	Fig. 1 Panel	References
Diatom	Araphid	*Fragilaria vaucheriae*		[5]
		Fragilariales *spp.*	23	[5]
		Odontidium *spp.*		[5]
		*Pseudostaurosira brevistriata*		[5]
		*Pseudostaurosira trainorii*		[5]
		*Punctastriata mimetica*	25	[15]
		*Staurosira construens binodis*		[5]
		*Staurosira construens venter*		[5]
		*Staurosirella leptostauron dubia*		[5]
		*Staurosirella leptostauron*		[5]
		*Staurosirella pinnata*		[5]
		Staurosirella *spp.*		[5]
		Tetracyclus spp.		[5]
	Asymmetric Biraphid	*Amphora coffeaeformis*		[5]
		Amphora *spp.*		[5]
		*Cymbella cymbiformis*	9	[12]
		Cymbella *spp.*	8,10	[5]
		Encyonema *spp.*		[5]
		Gomphoneis *spp.*		[5]
	Centric	unidentified	1a-1b,2,3	[5]
		Actinocyclus *spp.*		[5]
		*Alveophora americana*		[18]
		Aulacoseira *spp.*	4a-4c,6,7	[5]
		*Aulacoseira subarctica*	27	[16]
		*Aulacoseira tenella*	5a-5c	[11]
		*Aulacoseira ambigua*		[5]
		*Aulacoseira canadensis*		[5]
		*Aulacoseira pusilla*		[5]
		*Lindavia rossii*	11,16	[13]
		Pliocaenicus *spp.*	12,13,14,18	[5]
		Stephanodiscus *spp.*	15a-15b	[5]
		Chaetoceros *spp.*		[5]
		Semiorbis *spp.*	19,20	[5]
		unidentified	26	[5]
	Epithemioid	*Epithemia musculus*		[5]
	Eunotioid	Geissleria *spp.* (?)	24	[5]
		Craticula *spp.*	21,22	[5]
		Eunotia *spp.*		[5]
		Semiorbis *spp.*		[5]
	Monoraphid	Cocconeis *spp.*		[5]
		Planothidium apiculatum		[5]
		*Planothidium delicatulum*		[5]
	Nitzschioid	*Nitzschia fonticola*		[5]
	Surirelloid	*Surirella amphioxys*		[5]
	Symmetric Biraphid	Anomoeneis *spp.*		[5]
		*Anomoeneis sculpta*		[5]
		Navicula *spp.*		[5]
Phytolith	Bulliform flabellate	Poaceae	28	[4]
	Spheroid psilate	Broadleaf/Conifer	29, 31a-31b	[4]
	Spheroid ornate	Broadleaf/Conifer	30	[4]
	Spheroid granulate	Broadleaf/Conifer		[4]
	spheroid plicate	Broadleaf/Conifer		[4]
	Bilobate	Panicoideae/Poaceae	32a-32b	[4]
	Cross (polylobate)	Panicoideae/Poaceae		[4]
	Elongate entire (Type 1)	unidentified	33,34,35,36,39,43	[17]
	Elongate entire (Type 2)	unidentified	37	[17]
	Elongate arcuate	unidentified	38	[4]
	Polygonal tabular	unidentified	40,41,42,45,46	[4]
	Rondel	Poaceae	44	[4]
	Polyhedral (sclereid)	Broadleaf/Conifer	47,49	[4]
	Acicular (hair-like)	Poaceae	48	[4]
	Blocky	Broadleaf/Conifer	49,50,51	[4]
	Tracheary annulate	unidentified		[4]
Other	Sponge spicule	unidentified	52,53	[4]
	Undetermined	unidentified	54,55,56,57,58,59, 60	N/A

## 3 Data Description

The dataset includes 14 phytolith morphotypes and 45 identifiable diatom morphotypes >3µm. Phytolith morphotypes were described according to ICPN2.0 [4]. Phytoliths originated from both arboreal and grassland sources. Grassland morphotypes included bulliform flabellate (Fig. 1; 28), rondel (Fig. 1; 44), bilobates (Fig, 1; 32a-32b), and acicular (Fig. 1; 48) phytolith. Arboreal phytoliths included spheroids, both psilate (Fig. 1; 29, 31a-31b) and ornate (Fig. 1; 30), polyhedral sclereids (Fig. 1; 47,49), and blockies (Fig. 1; 49,50,51). Other phytolith morphotypes included elongate entire (Fig. 1; Type 1, 33,34,35,36,39,43; Type 2, 37), elongate arcuate (Fig. 1; 38), and polygonal tabulars (Fig. 1; 40,41,42,45,46).

Diatom morphotypes were evaluated based on the Database Diatoms of North America [5]. Diatoms were mostly centric and biraphid with some eunotioid and araphids present. Some centric diatoms were unidentifiable (Fig. 1; 1a-1b,2,3). Some were identifiable to the class or family level, including Aulacoseira spp. (Fig. 1; 4a-4c) and Stephanodiscus spp. (Fig. 1; 15a-15b), and some to the species level: A. tenella (Fig. 1; 5a-5c), L. rossii (Fig. 1; 11,16), C. tholiformis (Fig. 1; 17), and A. subarctica (Fig. 1; 27). Asymmetric Biraphid diatoms included Cymbella spp. (Fig. 1; 8,10) and C. cymbiformis (Fig. 1; 9). Symmetric Biraphid diatoms included Craticula spp. (Fig. 1; 21,22) and Geissleria spp. (Fig. 1; 24). Araphid diatoms were particularly small and were identified as Fragilariales spp. (Fig. 1; 24), with one species-level identification of P. mimetica (Fig. 1; 25). Finally, two Eunotioid diatoms were isolated, one non-identifiable (Fig. 1; 26) and the other identified as Semiorbis spp. (Fig. 1; 16,20). There were seven unidentified diatom morphotypes.

Other microremains included fragmented sponge spicules (Fig. 1; 52,53) and indeterminate microfossils (Fig. 1; 54,55,56,57,58,59,60).

## 4 Experimental Design, Materials and Methods

### 4.1 Experimental design

The ‘Bot-Meps’ Protocol [3] was followed for the sampling and slide preparation processes used to develop the presented dataset. Here is a brief summary of the major steps:

(1) A 5mm wide chisel was used to outline a 1 cm × 1 cm section as a sampling region. This was done in a fume hood onto a protective surface.
(2) The same chisel was used to separate the 1 × 1 cm sample from the rock matrix, at depths ranging from 2-4 cm, depending on the thickness of the specimen.
(3) The sample was placed in 1.5 mL Eppendorf tubes and ground to a fine powder using a needle tool. Between each sample the tools used were cleaned to prevent cross-contamination.
(4) Resulting powder was mounted onto glass slides with medium viscosity mounting oil and sealed.
(5) Slides were analyzed using a brightfield optical microscope with 40 and 100 x objective le5ses and 10 x eye lenses. Microphotographs were achieved using a Meiji Techno HD1500T microscope camera.

### 4.2 Materials

**Fig. 2:**
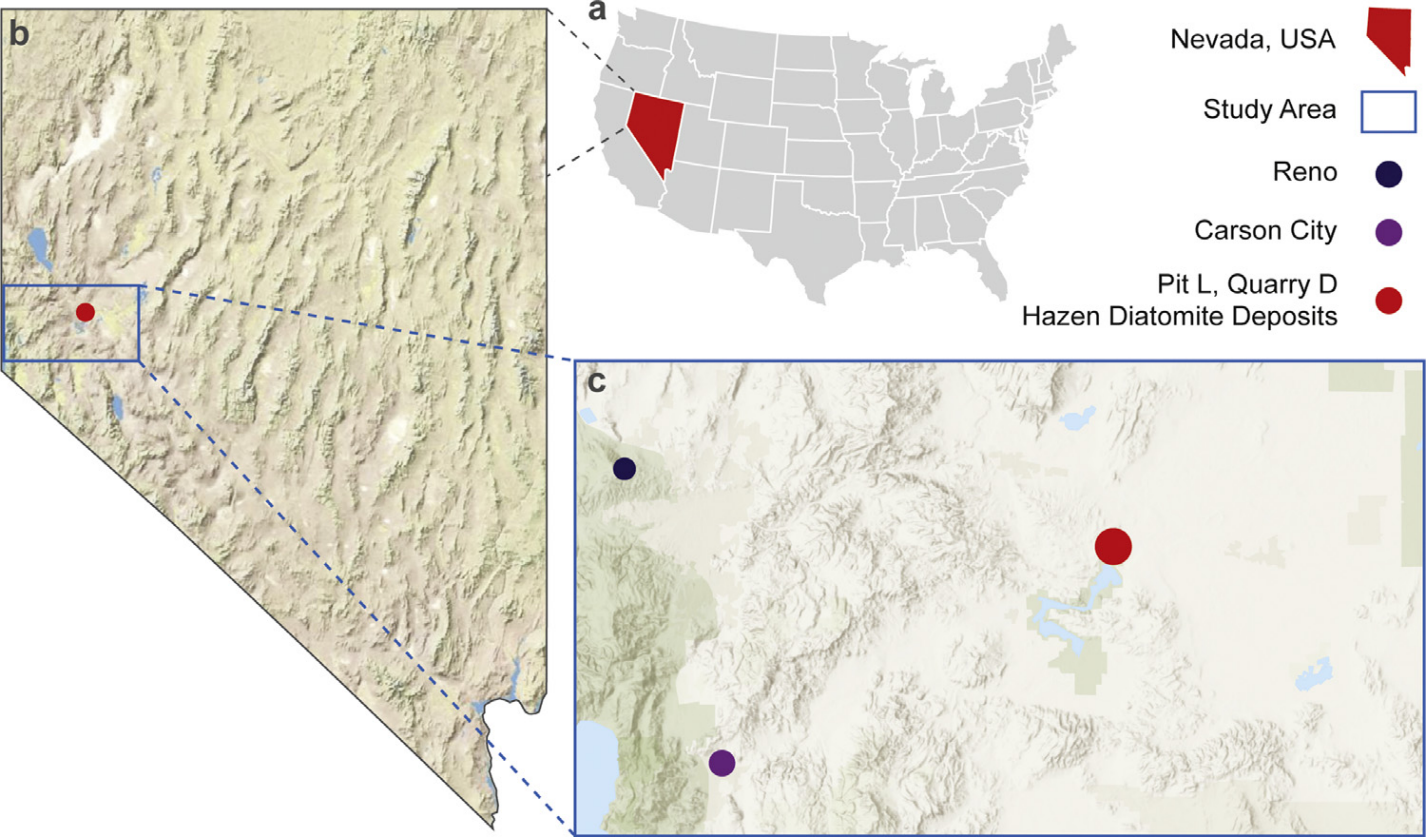
Location of the study area (c) within the state of Nevada (b) in the United States of America (a).

Diatomite samples came from Pit L, Quarry D, of the Hazen Diatomite Deposits, a 10.3 Myo Miocene paleolake (Fig. 2) from Northern Nevada [6,7]. Phytoliths and diatoms were extracted from diatomite samples at 20 cm intervals over 820 cm of section that captured adaptive evolution by G. doryssus [6], [8], [9], [10].

### 4.3 Methods

The samples were extracted and prepared following the ‘Bot-MEPS’ Protocol [3], though because our samples were free of carbonates and organic residues (Cerasoni, unpublished data) we did not follow the steps to remove those residues, nor did we need the heavy liquid flotation separation technique. The resulting ground samples were analyzed by brightfield optical microscopy at 40x and 100x magnification. The identification of each morphotype was carried out by matching with a high degree of confidence size, shape, surface texture and unique features to previously published databases and standards [11], [12], [13], [14], [15], [16], [17], [18]. All microfossils that did not match any known published diatom or phytolith morphotype were recorded as unidentified, but still presented here.

## Ethics Statement

All authors have read and follow the ethical requirements for publication in Data in Brief and confirming that the current work does not involve human subjects, animal experiments, or any data collected from social media platforms.

## CRediT authorship contribution statement

**Jacopo Niccolò Cerasoni:** Conceptualization, Investigation, Methodology, Software, Data curation, Visualization, Writing – original draft, Writing – review & editing. **Megan C. O'Toole:** Data curation, Methodology, Software, Writing – original draft. **Richa Patel:** Data curation, Methodology, Software. **Yoel E. Stuart:** Conceptualization, Investigation, Writing – review & editing.

## Data Availability

Botanical Microfossil Morphotypes - Hazen Diatomite Formation (Original data) (Figshare).

## References

[1] Bell M.A., Sadagursky M.S., Baumgartner J.V. (1987). Utility of lacustrine deposits for the study of variation within fossil samples. Palaios.

[2] Houseman M.D. (2004). Proceedings of the 39th Forum on the Geology of Industrial Minerals: Nevada Bureau of Mines and Geology Special Publication.

[3] O’Toole M.C., Stuart Y.E., Cerasoni J.N. (2022). Botanical Microfossil Extraction from Paleontological Sediments -. Bot-MEPS’ Protocol, protocols.io.

[4] International Committee for Phytolith Taxonomy (ICPT) (2019). International code for phytolith nomenclature (ICPN) 2.0. Ann. Botany.

[5] Spaulding (2021). Diatoms.org: supporting taxonomists, connecting communities. Diatom Research.

[6] Bell M.A., Travis M.P., Blouw D.M. (2006). Inferring natural selection in a fossil threespine stickleback. Paleobiology.

[7] J.N. Cerasoni, M.A. Bell, Y.E. Stuart, Geomorphology of Miocene diatomite deposits from Hazen, Nevada, with stratigraphy of a stickleback (Gasterosteus doryssus) fossil fish sequence. PaleoBios (in review).

[8] Gene H., Bell M.A., Travis M.P. (2008). Evolution toward a new adaptive optimum: phenotypic evolution in a fossil stickleback lineage. Evolution.

[9] Stuart Y.E., Travis M.P., Bell M.A. (2020). Inferred genetic architecture underlying evolution in a fossil stickleback lineage. Nature Ecol. Evol..

[10] Voje K.L., Bell M.A., Stuart Y.E. (2022). Evolution of static allometry and constraint on evolutionary allometry in a fossil stickleback. J. Evol. Biol..

[11] Siver P.A., Kling H. (1998). Morphological observations of Aulacoseirausing scanning electron microscopy. Can. J. Bot..

[12] Patrick R.M., Reimer C.W., W C. (1975). The Diatoms of the United States, exclusive of Alaska and Hawaii. V. 2 Monogr. Acad. Natural Sci. Philadelphia.

[13] Nakov T., Guillory W., Julius M., Theriot E., Alverson A. (2015). Towards a phylogenetic classification of species belonging to the diatom genus Cyclotella (Bacillariophyceae): transfer of species formerly placed in Puncticulata, Handmannia, Pliocaenicus and Cyclotella to the genus Lindavia. Phytotaxa.

[14] Håkansson H., Kling H. (1990). The current status of some very small freshwater diatoms of the genera Stephanodiscus and Cyclostephanos. Diatom Res..

[15] Morales E.A. (2005). Observations of the morphology of some known and new fragilarioid diatoms (Bacillariophyceae) from rivers in the USA. Phycol. Res..

[16] Gibson C.E., Anderson N.J., Haworth E.Y. (2003). Aulacoseira subarctica: taxonomy, physiology, ecology and palaeoecology. Eur. J. Phycol..

[17] Runge F. (1999). The opal phytolith inventory of soils in central Africa—quantities, shapes, classification, and spectra. Rev. Palaeobotany Palynol..

[18] Usoltseva M., Kociolek J.P., Khursevich G. (2013). Three new species of *Alveolophora* (Aulacoseiraceae, Bacillariophyceae) from Miocene deposits in western North America. Phycologia.

